# Development and validation of circulating tumor cells signatures for papillary thyroid cancer diagnosis: A prospective, blinded, multicenter study

**DOI:** 10.1002/ctm2.142

**Published:** 2020-08-12

**Authors:** Siyuan Xu, Jingning Cheng, Bojun Wei, Yang Zhang, Yang Li, Zongmin Zhang, Yang Liu, Ye Zhang, Rui Zhang, Kai Wang, Xiaolei Wang, Shaoyan Liu, Ying Huang, Zhengang Xu, Jie Liu

**Affiliations:** ^1^ Department of Head and Neck Surgical Oncology National Cancer Center/National Clinical Research Center for Cancer/Cancer Hospital Chinese Academy of Medical Sciences and Peking Union Medical College Beijing P. R. China; ^2^ Department of Otorhinolaryngology China‐Japan Friendship Hospital Beijing P. R. China; ^3^ Department of Thyroid and Neck Surgery Beijing Chaoyang Hospital Capital Medical University Beijing P. R. China; ^4^ Department of Endocrinology Peking University First Hospital Beijing P. R. China; ^5^ Department of General Surgery Hebei Petro China Central Hospital Langfang P. R. China; ^6^ Department of Radiation Oncology National Cancer Center/National Clinical Research Center for Cancer/Cancer Hospital Chinese Academy of Medical Sciences and Peking Union Medical College Beijing P. R. China; ^7^ Department of Ultrasound National Cancer Center/National Clinical Research Center for Cancer/Cancer Hospital Chinese Academy of Medical Sciences and Peking Union Medical College Beijing P. R. China

Dear editor,

Thyroid nodules are common and the major point of their clinical evaluation rests with the differentiation of thyroid cancer.^1^, ^2^, ^3^ Herein, we present a two‐step study to develop and validate an effective model based on circulating tumor cells (CTCs) signatures for papillary thyroid cancer (PTC) diagnosis.

A total of 532 subjects were clinically evaluated and 220 of which were included in the final analysis (Figure 1A). In the first step, 70 patients with PTC from a single hospital and 20 healthy subjects formed the test group. Multi‐markers reported in the literatures that were related to malignancy and thyroid epithelium (CK19, Survivin, Galectin‐3, Tg, and TSHR) were evaluated and selected to form a most effective combination for the diagnostic model. Next, the model was evaluated in the validation group that was consisted of 150 participants from four different hospitals in China who had one or more thyroid nodules that yielded an ultrasound diagnosis of Thyroid Imaging, Reporting and Data System^4^, ^5^ (TI‐RADS) 4 or 5 and measured 0.3‐2.0 cm long in diameter. Postoperative pathology was considered as the reference standard and only patients who received surgery and had definite pathological reports were enrolled in the final analysis. Thyroid nodule was excluded by ultrasound in healthy subjects.

**FIGURE 1:**
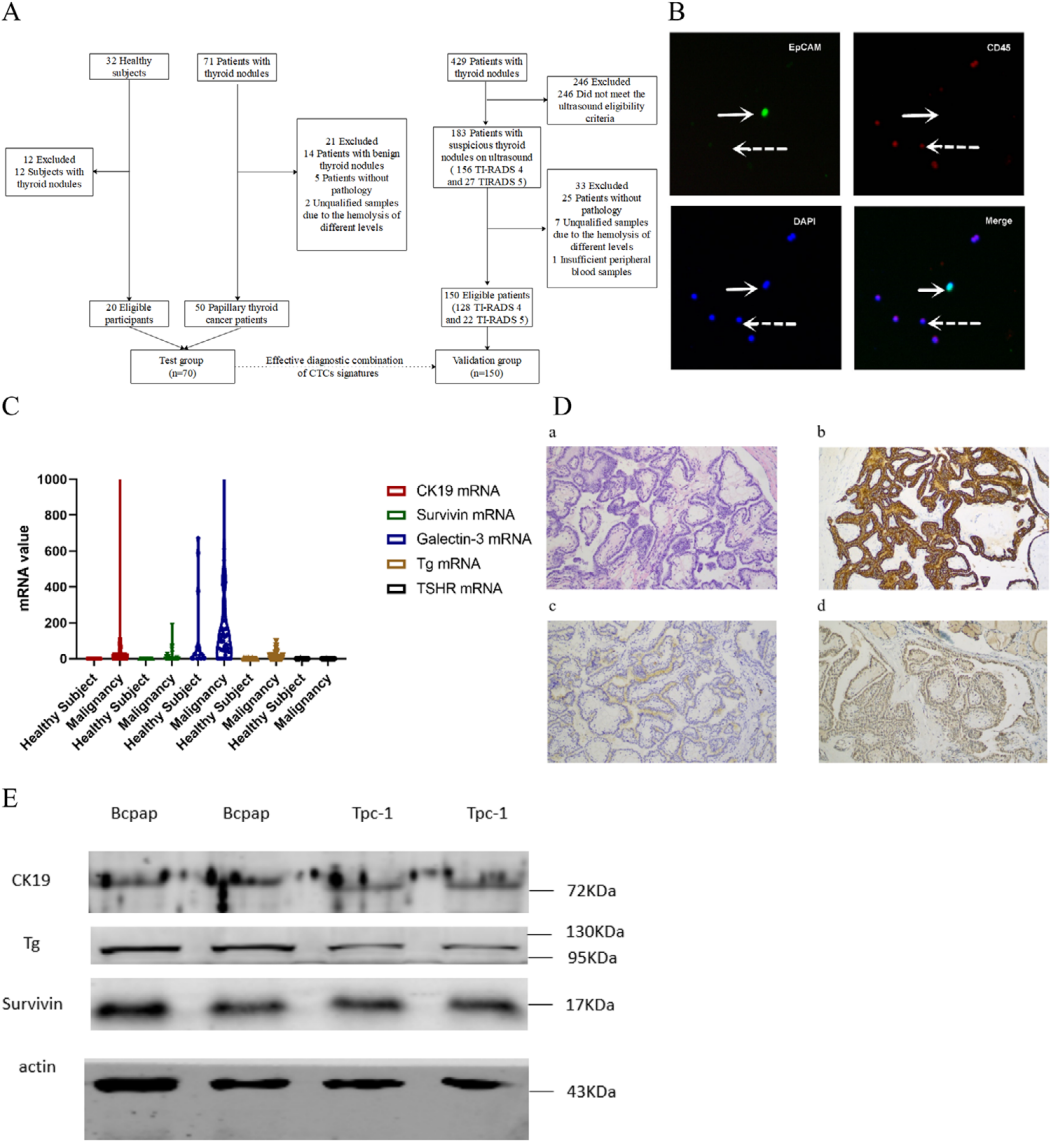
Flow chart of the study and diagnostic model establishing. A, Flow chart of the study. B, Fluorescence staining of EpCAM, CD45 and DAPI after immunomagnetic enrichment of a CTC model (100 PTC cells added into white blood cells separation from 4 ml peripheral blood from a healthy subject) (CTCs, white solid arrow; white blood cells, white dotted arrow). C, CK19, Survivin, Galectin‐3, Tg, and TSHR mRNA levels of healthy subjects (n = 20) and patients with PTC (n = 50) in the test group. D, HE (a) and immunohistochemical staining for CK19 (b), Tg (c), and Survivin (d) of a PTC patient enrolled in the test group. E, Expression of markers in cell lines of PTC by western blot

The process of CTCs separation and enrichment included CD45 negative (Dynabeads™ M‐450 CD45 pan Leukocyte, #11153D, Invitrogen by Thermo Fisher Scientific, USA) and EpCAM positive (#161.02, Invitrogen by Thermo Fisher Scientific, USA) immunomagnetic selections. The reliability of CTCs separation and enrichment was verified with fluorescence staining in a cell line model (100 PTC cells added into white blood cells separation from 4 mL peripheral blood from a healthy subject; Figure 1B). mRNA was isolated from the cell fractions (#61021, Invitrogen by Thermo Fisher Scientific, USA) and cDNA was then reverse transcribed with 12 μL of mRNA (total 20 μL) using the Sensiscript RT Kit (QIAGEN #205213, Germany). mRNA levels of CK19, Survivin, Galectin‐3, Tg, and TSHR were analyzed by the QuantiNova SYBR Green PCR Kit (QIAGEN #208056, Germany; Thermo Fisher Scientific, USA). Three independently isolated RNA samples were measured to determine the threshold cycle values (CT). mRNA level of these markers were measured with relative expression level (R_Q_) and presented after normalization against the reference, β‐actin, and was calculated with 2^–ΔΔCT^ method (ΔCt = Ct (target) – Ct (β‐actin), ΔΔCT = ΔCt‐13, R_Q _= 2^–ΔΔCT^,). The values were presented as the mean ± standard deviation (SD).

Of all 220 participants recruited in the study, the median age was 43 years (range, 19‐72 years) and 77.7% were female (171 participants). Among 200 patients with thyroid nodules, the median size of the nodules was 1.0 cm (range, 0.3‐5.5 cm) and 57.5% had a tumor ≤1 cm. According to the postoperative pathology, 163 patients had PTC, of which 59 and 20 patients had central compartment and lateral neck lymph nodes metastasis, respectively. In the test group (50 patients and 20 healthy subjects), the median mRNA levels of the markers were significantly higher in patients than in healthy controls except for TSHR (Figure 1C). CK19 and Survivin mRNA was not detected in any healthy subject so positive result of the two markers was defined as detectable mRNA level, Tg and Galectin‐3 mRNA were detected in 35% and 50% healthy subjects, respectively. Based on the ROC curves, a cutoff value of 33.05 ng/μg was selected for Galectin‐3, with a sensitivity of 78% and a specificity of 70%, while a cutoff value of 5.71 ng/μg for Tg, giving an optimal sensitivity of 42% and a specificity of 100%. The area under curve (AUC) of CK19, Survivin, Galectin‐3, and Tg were 0.740, 0.620, 0.712, and 0.668, respectively.

After evaluating different combinations of the markers, (Supporting Information 1) CK19, Survivin, and Tg were selected to form a diagnostic model, when positive result was recognized when any marker was positive its positive predictive value (PPV), negative predictive value (NPV), sensitivity, specificity, and AUC value were 100%, 66.7%, 80%, 100%, and 90% respectively, and the diagnostic model correctly classified 60 of 70 subjects, yielding an overall accuracy of 85.7%. The expression of selected markers (CK19, Survivin, and Tg) was verified with immunohistochemical analysis in formalin‐fixed paraffin‐embedded postoperative specimen of PTC patients (Figure 1D) and western blot in two cell lines of PTC (Figure 1E), and then these markers were evaluated in the validation group.

In the validation cohort (n = 150), the median mRNA levels of CK19, Survivin, and were significantly higher in patients with PTC than in patients with benign thyroid nodules (*P* < .05) (Figure 2A). The clinical sensitivity, specificity, PPV, and NPV of the diagnostic model were 82.3%, 81.1%, 93.0%, and 60.0%, respectively, with a diagnostic accuracy of 82.0%, and the AUC for predicting malignancy was 0.857 (95% CI, 0.796‐0.919). For detailed performance of individual markers, the highest sensitivity of single marker (Tg 59/113) was 52.2%, which was significantly lower than the diagnostic model of 3‐marker combination. All patients with positive Survivin (n = 41) had malignant pathology (PPV = 100%) with a sensitivity of 36.3%, which was consistent with the results of the test group. (Figure 2B)

**FIGURE 2:**
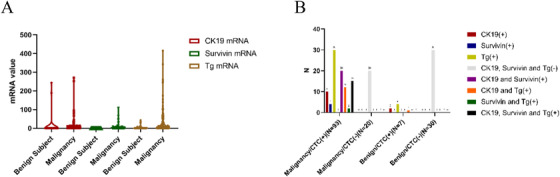
Validation of the diagnostic model in a prospective cohort (n = 150). A, CK19, Survivin, and Tg mRNA levels of benign and malignant patients in the validation group. B, Detailed diagnostic performance of the diagnostic model in the validation group

In conclusion, the diagnostic model based on three CTCs signatures established in the study has a high diagnostic performance for patients with suspicious thyroid nodules, multi‐marker analysis significantly improved sensitivity comparing with single marker analysis. Moreover, it is discovered in this study that the CTCs signature Survivin had a potential value for confirming PTC diagnosis. (PPV = 100%).

## CONFLICT OF INTEREST

The authors have no conflicts of interest to disclosure.

## STATEMENT OF ETHICS

The study was approved by the ethic committee of Cancer Hospital, Chinese Academy of Medical sciences.

## AUTHORS’ CONTRIBUTIONS

Siyuan Xu and Jingning Cheng participated in the design of the study, data collection, and paper writing. Bojun Wei and Yang Zhang participated in the data collection and helped to draft the manuscript. Yang Li and Rui Zhang participated in the data analysis and validation. Zongmin Zhang, Yang Liu, and Kai Wang participated in the data collection and quality control of data. Ye Zhang and Ying Huang participated in the statistical analysis. Xiaolei Wang and Shaoyan Liu participated in the manuscript review. Jie Liu and Zhengang Xu participated in the design of the study and helped to revise the manuscript. All authors read and approved the final manuscript.

## Supporting information

Supporting Information.
