# Supplementary material for: Development and validation of circulating tumor cells signatures for papillary thyroid cancer diagnosis: A prospective, blinded, multicenter study
Source: Clin Transl Med. 2020 Aug 12;10(4):e142. doi: 10.1002/ctm2.142 (PMC7423182; doi:10.1002/ctm2.142)
Supplement: Supplementary file 1 — Supporting Information. [file CTM2-10-e142-s001.pdf]

Supplementary 1. Diagnostic performance of different combinations of mRNA models in the test group (n=70)

	Sensitivity (%)	Specificity (%)	NPV (%)	PPV (%)	Accuracy (%)	-LR	+LR	AUC
CK19/Tg	74.00	100.00	60.60	100.00	81.43	0.26	Inf	0.870
CK19/Survivin	62.00	100.00	51.28	100.00	72.86	0.38	Inf	0.810
CK19/Galectin-3	86.00	70.00	80.95	87.76	81.43	0.20	2.87	0.852
Tg/Survivin	52.00	100.00	45.45	100.00	65.71	0.48	Inf	0.760
Tg/Galectin-3	90.00	70.00	73.68	88.24	84.29	0.14	3.00	0.863
Survivin/Galectin-3	78.00	70.00	56.00	86.67	75.71	0.31	2.60	0.776
CK19/ Survivin/ Tg*	80.00	100.00	66.67	100.00	85.71	0.20	Inf	0.900
CK19/Tg/Galectin-3	94.00	70.00	82.35	88.68	87.14	0.09	3.13	0.931
CK19/Survivin/Galectin-3	86.00	70.00	66.67	87.76	81.43	0.20	2.87	0.873
Tg/Survivin/Galectin-3	90.00	70.00	73.68	88.24	84.29	0.14	3.00	0.863
CK19/Tg/Survivin/Galectin-3	94.00	70.00	82.35	87.68	87.14	0.09	3.13	0.940

*This combination of signatures was selected as the most highly effective diagnostic model with 100% PPV and a high NPV.

For Re
